# Editorial: Enhancing crop resilience to salt stress

**DOI:** 10.3389/fpls.2026.1780845

**Published:** 2026-01-16

**Authors:** Analía Susana Llanes, Vivek Ambastha, Raoudha Abdellaoui

**Affiliations:** 1 Universidad Nacional de Río Cuarto (UNRC), Córdoba, Argentina; 2 Consejo Nacional de Investigaciones Científicas y Técnicas (CONICET), Instituto de Investigaciones Agrobiotecnológicas, INIAB, (CONICET-UNRC), Córdoba, Argentina; 3 Department of Biology, Washington University in St. Louis, St. Louis, MO, United States; 4 Laboratory of Rangeland Ecosystems and Valorization of Spontaneous Plants and Associated Microorganisms (LR16IRA03), Arid Regions Institute, University of Gabes, Medenine, Tunisia

**Keywords:** engineering salt-tolerant crops, plant-microorganisms interaction, salt stress mitigation, salt tolerance criteria, salt-tolerance functional bases

## Introduction

Soil salinization represents one of the most severe abiotic constraints on global agricultural productivity, currently affecting nearly 20% of cultivated land and expected to expand substantially by 2050 (Mann et al., 2023; Zahid et al., 2025). Salinity originates from a combination of natural processes and human activities, including inadequate irrigation practices, excessive fertilizer application, climate-driven changes in water availability, and seawater intrusion (Kumar and Sharma, 2020). Salinity rarely operates as a standalone stress; instead, it interacts dynamically with environmental variables such as temperature, precipitation, and evaporative demand, generating complex spatiotemporal patterns that intensely shape plant performance and yield responses (Hassani et al., 2021).

At the plant level, salinity imposes a dual constraint through osmotic stress, which limits water uptake, and ionic stress arising from the excessive accumulation of toxic ions such as sodium and chloride. Together, these stresses disrupt cellular homeostasis, impair photosynthetic efficiency, limit nutrient acquisition, and ultimately reduce growth and productivity (Hu et al., 2025). As a consequence, soil salinization has transitioned from a localized agronomic concern to a global challenge with far-reaching implications for food security, ecosystem functioning, and the long-term sustainability of agricultural systems. Addressing this challenge requires the coordinated development of salt-tolerant crops alongside management strategies that enhance agroecosystem resilience. This Research Topic contributes to this effort by consolidating recent advances in our understanding of plant adaptive responses to salinity and by highlighting strategies to translate this knowledge into crop improvement. The compiled studies assembled physiological, biochemical, molecular, and ecological scales, collectively illustrating how salt tolerance emerges from the integration of multiple regulatory processes rather than from isolated traits. Importantly, the contributions emphasize that salinity stress must be interpreted within the broader environmental context in which crops develop. Evidence from field-based and long-term investigations demonstrates that salt accumulation and its impact on plant performance are strongly modulated by seasonal fluctuations in temperature, rainfall, soil moisture, and irrigation regimes. In both coastal and inland saline–alkali systems, adverse conditions during early developmental stages, such as low temperatures and water deficit during germination, can amplify salinity effects, whereas periods of increased rainfall may facilitate salt leaching and partial recovery during later growth phases.

Taken together, the studies presented in this Research Topic reinforce the view of salinity as a dynamic, multifactorial stress that interacts with other abiotic constraints rather than acting in isolation. From an agronomic and ecological perspective, these findings underscore the necessity of integrated approaches that combine informed crop selection with adaptive soil and water management to mitigate salinity impacts and sustain crop productivity under increasingly variable environmental conditions.

## Halophytes as model for to enhance crop resilience to salt stress and saline soil utilization

Halophytes represent a biologically and ecologically valuable resource for the sustainable use and rehabilitation of saline soils. Although they constitute less than 1% of terrestrial plant species, halophytes possess highly specialized mechanisms that enable survival and growth under salinity levels ranging from 50 to more than 500 mM NaCl (Flowers and Colmer, 2015; Mann et al, 2023). These mechanisms include efficient ion compartmentalization, osmotic adjustment, and the maintenance of photosynthetic integrity, as illustrated by several studies included in this Research Topic. Therefore, Conversa et al. investigated the morpho-biophysiological traits, mineral nutrition, and antioxidative responses of a pinnatifid population of *Cakile maritima* from the Apulia region grown under increasing NaCl concentrations. Their results confirmed the classification of this species as a sodium-including halophyte, exhibiting optimal growth at moderate salinity (~100 mM NaCl). Notably, plants maintained leaf sodium concentrations compatible with photosynthetic activity even under high salinity by reallocating excess Na^+^ to the stems. The stable uptake of essential cations such as K^+^, Ca²^+^, and Mg²^+^ further indicates an efficient ion homeostasis under saline conditions. Similarly, Kachout et al. conducted field studies on *Atriplex hortensis* that demonstrated its strong phytoremediation potential in saline environments. Despite reductions in biomass, plants maintained stable water status and stomatal conductance. Moreover, cultivation under saline conditions resulted in measurable decreases in soil electrical conductivity, confirming the species’ capacity for phytodesalinization. These traits underscore the value of halophytes in integrated strategies that combine forage production with soil rehabilitation.

Finally, Ma et al. examined the responses of the ice plant, *Mesembryanthemum crystallinum*, to combined salinity and alkalinity stress. By evaluating growth, mineral composition, and phytochemical profiles across stress intensities, the authors highlighted the productive potential of saline agriculture. Moderate saline–alkali stress enhanced antioxidant activity, soluble sugars, vitamins, flavonoids, and enzymatic defense systems, whereas severe stress induced ionic toxicity, oxidative damage, and growth inhibition. These findings underscore the importance of defining optimal stress thresholds that maximize nutritional and functional benefits while minimizing yield penalties, and they highlight the potential of ice plant cultivation as a valuable vegetable crop for saline–alkali environments.

Collectivelly, these studies reinforce the concept of halophytes as multifunctional biological resources capable of sustaining productivity while contributing to the remediation of salt-affected soils. By elucidating species-specific physiological thresholds and adaptive strategies, the contributions presented here provide a robust framework for advancing saline agriculture and ecosystem restoration. Harnessing the inherent resilience of halophytes offers a promising pathway toward productive land use in saline environments, aligning agricultural innovation with ecological sustainability.

## Molecular, genomic and proteomic mechanisms of crop resilience to salt stress

Recent advances in transcriptomic, proteomic, and genomic approaches have significantly improved our understanding of crop resilience to salt stress. In this context, Cao et al. explored the transcriptomic profiling of *Lactuca indica* under seawater irrigation revealed coordinated regulation between above- and belowground tissues, involving genes related to photosynthesis (LHC), wax biosynthesis (CER1), cytochrome P450 enzymes, and nitrogen metabolism (NR, NRT2, NirA), providing a theoretical foundation for seawater-based agricultural systems. Futhermore, transcriptional regulation plays a central role in mediating salinity responses. In this Research Topic, comprehensive genomic analyses of plant-specific transcription factors that regulate plant growth, development, and stress response were performed. Wang et al. identified key hub genes in mung bean involved in hormone signaling and MAPK pathways, underscoring their role in salt stress adaptation. The study provide molecular insights that may facilitate the development of salt-tolerant mung bean varieties through molecular breeding. They findings also offer a foundation for future functional studies aimed at improving crop resilience under salt stress conditions. Similarly, Yin et al. elucidated the molecular mechanisms underlying salt tolerance in rice. The functional characterization of the MIKC-type MADS-box gene OsMADS31 in rice demonstrated that this regulator enhances salt tolerance by activating antioxidant enzymes, reducing oxidative damage, and modulating stress-responsive gene networks. Loss-of-function mutants exhibited reduced survival, impaired growth, and elevated ROS accumulation, whereas overexpression lines maintained normal development under salt stress conditions.

Zhang et al. examined the proteomic comparisons between salt-tolerant and salt-sensitive potato cultivars. Their analysis revealed that salt tolerance is associated with coordinated activation of redox homeostasis, carbohydrate metabolism, phytohormone signaling, and protein turnover pathways. In contrast, salt-sensitive cultivars exhibit fragmented and less efficient stress response mechanisms.

Together, these studies offer new insights into the molecular targets and proteomic mechanisms that govern salt tolerance. In addition, they identify potential candidate genes for breeding and genetic engineering aimed at enhancing crop resilience to salt stress.

## Biological strategies for enhancing crop resilience to salt stress

Biological solutions are increasingly recognized as sustainable tools for mitigating salt stress responses. The review of Nguyen et al. focusing on rice cultivation in the Vietnamese Mekong Delta highlight the potential of plant growth-promoting rhizobacteria to enhance salt tolerance through improved nutrient uptake, osmotic adjustment, and activation of stress-responsive genes. However, limited long-term field studies and restricted international dissemination of regional research remain major constraints to large-scale adoption.

The review by Wang et al. extends the salinity resilience framework to ornamental horticulture, a sector increasingly challenged by the use of saline and reclaimed water resources. By synthesizing current knowledge on the impacts of salinity on aesthetic quality, growth, photosynthetic performance, and ion homeostasis, the authors provide a valuable foundation for informed species selection and sustainable irrigation management. However, the scarcity of empirical data for many commercially important ornamental species highlights a significant research gap, underscoring the need for standardized assessment criteria and integrated evaluations that link physiological responses with visual quality.

Collectively, these studies reinforce the notion that enhancing resilience to salt stress requires a holistic approach that integrates biological solutions, species-specific tolerance assessments, and context-dependent management strategies. Advancing such integrated frameworks will be essential for sustaining productivity and quality across diverse agricultural and horticultural systems under increasing salinity pressure.

## Intraspecific variability, breeding, and predictive approaches

Salinity tolerance is highly dependent on genotype and developmental stage. Martroberardino et al. combined time-to-event germination modeling with stress tolerance indices and multivariate analyses to investigate intraspecific variability in *Camelina sativa* under salinity stress during germination and early seedling growth, and to evaluate the extent to which early-stage traits can anticipate later performance. Their results revealed pronounced intraspecific diversity at early developmental stages, reflecting distinct adaptive strategies that do not necessarily translate into superior performance at subsequent growth phases. These findings highlight the limitations of relying solely on germination-based traits and underscore the importance of multistage phenotyping approaches in breeding programs aimed at improving salt tolerance.

Comprehensive multifactorial analyses that integrate annual environmental variability with crop physiological performance under saline stress are very important but scarce. However, in this Research Topic, Feng et al. conducted a two-year field study on naturally saline-alkaline coastal soils, examining how interannual variations in water availability, temperature regimes, and soil salinity jointly regulate cotton photosynthetic performance and yield formation. The study systematically characterized (i) the seasonal dynamics of precipitation and temperature, (ii) the spatiotemporal fluctuations of soil moisture and salinity, and (iii) their cumulative effects on key stages of cotton development. They quantified a comprehensive set of photosynthetic and growth-related characteristics, including canopy photosynthetic and respiration rates, chlorophyll content, leaf area index, and photoassimilate distribution, to elucidate the mechanistic links between combinations of environmental stress and yield limitations. By integrating the temporal and spatial dimensions of water-salt-heat interactions, this work provides a predictive physiological framework that supports genotype-based management and breeding strategies aimed at optimizing cotton productivity under saline-alkaline conditions.

Innovative approaches combining physiology with predictive modeling further expand strategies for salinity management. In this context, Shang et al. finded an innovative method that can not only effectively increase the concentration of the functional compounds in roots under salt stress, but also significantly promote its root biomass, thereby increasing both the yield and the quality of the medicinal and edible organ. They demostrated that in *Glycyrrhiza uralensis*, exogenous application of lanthanum nitrate mitigates salt-induced growth inhibition while enhancing root biomass and the accumulation of pharmacologically active compounds.

These studies collectively reinforce the concept that integrating physiological measurements with advanced predictive modeling enables a more accurate assessment of plant responses to salt stress. These integrated approaches enable a more accurate and comprehensive understanding of plant behavior under saline conditions. Moreover, they provide innovative tools for precision cultivation and supports the development of crops with enhanced salinity resilience.

## Conclusions and perspectives

Collectively, the contributions presented in this Research Topic offer a comprehensive, multi-scale understanding of plant responses to salt stress, integrating environmental factors with physiological, molecular, and agronomic perspectives. These contributions emphasize the key functional bases of salt tolerance, including photosynthetic maintenance, source-sink coordination, antioxidant defense, ionic homeostasis, transcriptional regulation, and beneficial plant-microorganism interactions. By linking mechanistic knowledge with applied breeding and management strategies, these contributions drive the identification of key traits, biological solutions, and predictive approaches needed to enhance crop resilience under saline conditions and support the development of more sustainable agricultural systems. As soil salinization intensifies under the combined pressures of climate change and agricultural intensification, interdisciplinary and integrative research efforts such as those highlighted here will be indispensable. Translating this knowledge into resilient crop varieties and adaptive production systems will be crucial to maintaining agricultural productivity, stabilizing yields, and ensuring food security in salinity-affected regions worldwide. Future research should focus on integrating multiomics approaches with high-resolution phenotyping and machine learning tools to better predict plant performance under diverse and fluctuating salt stress conditions. In addition, expanding studies on interactions among genotype, saline environment, and biological solutions, and translating these findings into field-based applications, will be essential for accelerating the development of salt-resilient crops adapted to real-world agricultural systems.
