# Correction: Editorial: Enhancing crop resilience to salt stress

**DOI:** 10.3389/fpls.2026.1802232

**Published:** 2026-07-20

**Authors:** Analía Susana Llanes, Vivek Ambastha, Raoudha Abdellaoui

**Affiliations:** 1 Universidad Nacional de Río Cuarto (UNRC), Córdoba, Argentina; 2 Consejo Nacional de Investigaciones Científicas y Técnicas (CONICET), Instituto de Investigaciones Agrobiotecnológicas, INIAB, (CONICET-UNRC), Córdoba, Argentina; 3 Department of Biology, Washington University in St. Louis, St. Louis, MO, United States; 4 Laboratory of Rangeland Ecosystems and Valorization of Spontaneous Plants and Associated Microorganisms (LR16IRA03), Arid Regions Institute, University of Gabes, Medenine, Tunisia

**Keywords:** engineering salt-tolerant crops, plant-microorganisms interaction, salt stress mitigation, salt tolerance criteria, salt-tolerance functional bases

Authors “Vivek Ambastha and Raoudha Abdellaoui” were omitted as authors in the published article. The correct author list reads:

“Analía Susana Llanes^1,2^*, Vivek Ambastha^3^, Raoudha Abdellaoui^4^”

“^1^Universidad Nacional de Río Cuarto (UNRC), Córdoba, Argentina

^2^Consejo Nacional de Investigaciones Científicas y Técnicas (CONICET), Instituto de Investigaciones Agrobiotecnológicas, INIAB, (CONICET-UNRC), Córdoba, Argentina

^3^Department of Biology, Washington University in St. Louis, St. Louis, MO, United States.

^4^Laboratory of Rangeland Ecosystems and Valorization of Spontaneous Plants and Associated Microorganisms(LR16IRA03), Arid Regions Institute, University of Gabes, Medenine, Tunisia”.

The **Author contributions** statement has been corrected to read:

“AL: Writing – review & editing. VA: Writing – review & editing. RA: Writing – review & editing.”

The **Acknowledgements** statement has been added to read:

“The guest editors of this Research Topic would like to thank Prof. Chun-Ming Liu, Chief Editor of Frontiers in Plant Science. Our thanks also go to the Journal Specialists of Frontiers in Plant Science. We cannot forget also to thank all the authors that contributed with their research and review articles to achieve the goal of our Research Topic.”

The original version of this article has been updated.

